# Mechanisms of Degradation and Damage in GaAs PHEMT Low-Noise Amplifier Under Ultra-Short Microwave Pulses

**DOI:** 10.3390/mi17091054

**Published:** 2026-09-03

**Authors:** Tongxin Guan, Liang Cheng, Zhiyuan Zhang, Yingjian Cao, Yu Wang, Guo Liu

**Affiliations:** 1 School of Electronic Science and Engineering, University of Electronic Science and Technology of China, Chengdu 611731, China; tongxinguan_uestc@163.com (T.G.); 202422020119@std.uestc.edu.cn (L.C.); zzy39_uestc@163.com (Z.Z.); cyj18883196024@163.com (Y.C.); wangyu.uestc.ese@gmail.com (Y.W.); 2 The 13th Research Institute of China Electronics Technology Group Corporation, Shijiazhuang 050051, China

**Keywords:** GaAs pseudomorphic high-electron-mobility transistors (pHEMT) low-noise amplifier, ultra-short microwave pulse, damage effects

## Abstract

Ultra-short microwave pulses with durations of 1 ns or less can go through a PIN limiter with low attenuation before the limiter responds and can therefore be directly injected into the subsequent low-noise amplifier (LNA). Based on this consideration, this paper reports the first investigation of degradation and damage in the GaAs pseudomorphic high-electron-mobility transistors (pHEMT) LNA induced by high-power ultra-short microwave pulses. The effects of pulse power, frequency, and repetition rate on the degradation and damage mechanisms of the LNA are investigated. The results show that, when exposed to a 45 dBm ultra-short microwave pulse at 2 GHz, the region beneath the transistor gate on the drain side experiences the most rapid burnout within 43 ns at a repetition rate of 200 MHz. Further analysis reveals that the negative half-cycle of the high-power microwave pulse depletes the electron concentration in the transistor. Consequently, excessively high repetition rates of high-power ultra-short microwave pulses are less likely to induce transistor burnout. Furthermore, the gain of the LNA is affected by both the electron concentration and the electron mobility in the transistor. The injected high-power ultra-short microwave pulses disturb the electron concentration, while the temperature rise reduces electron mobility. As a result, the transistor performance degrades, leading to a decrease in the LNA gain.

## 1. Introduction

**Figure 1:**
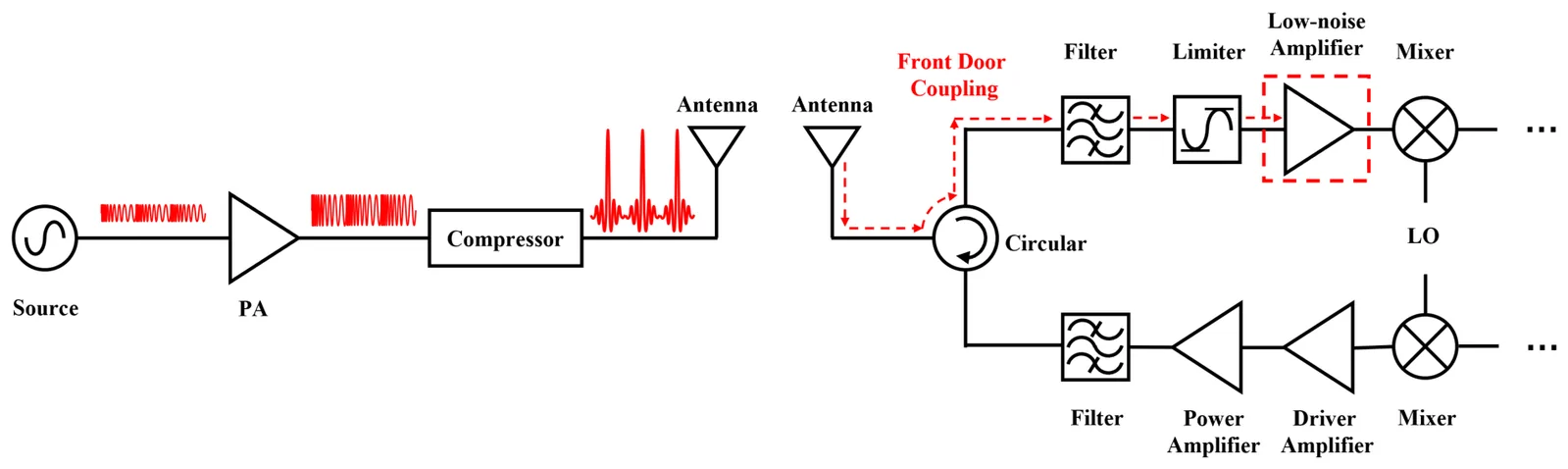
Schematic diagram of high-power ultra-short microwave pulses damage the LNA of RF receiver.

**Figure 2:**
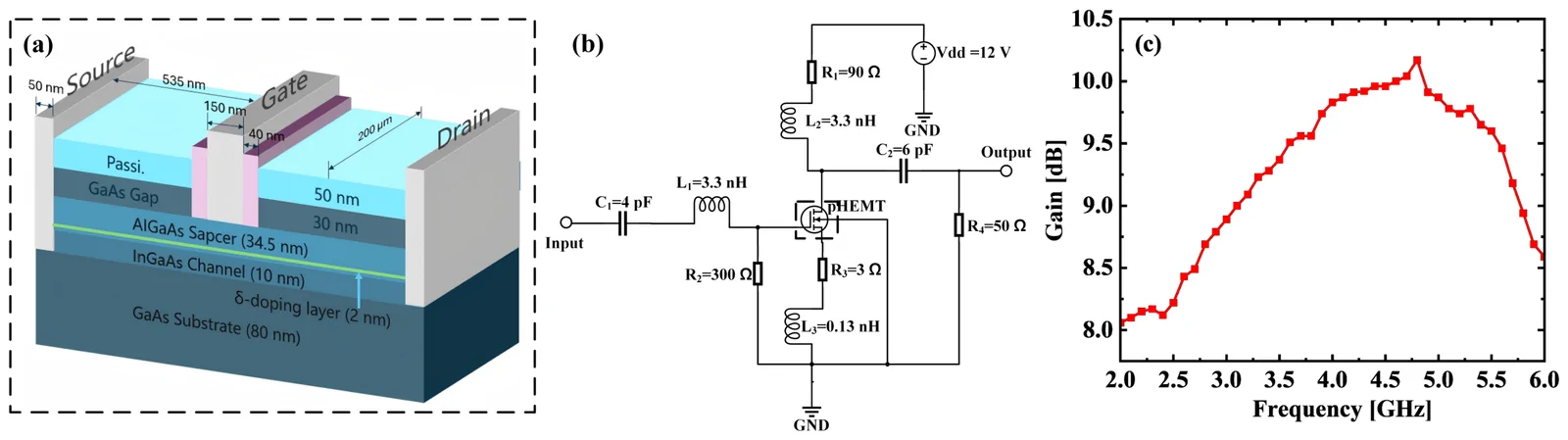
(**a**) The structure of the GaAs pHEMT, (**b**) the circuit diagram of LNA and (**c**) gain of the LNA.

Low-noise amplifiers (LNAs) are widely employed in radio-frequency (RF) receivers owing to their high sensitivity and low noise figure [1,2]. They amplify weak signals captured by the receiving antenna and contribute to maintaining a low overall noise level for the receiver system. GaAs-based pseudomorphic high-electron-mobility transistors (pHEMTs) are widely used in the design of LNAs due to their high electron mobility, high operating frequency and high efficiency [3,4,5].

The LNA is typically the first active device following the antenna in an RF receiver. As a result, it is vulnerable to damage caused by high-power microwave (HPM) injection. When HPM is injected into a GaAs-based pHEMT LNA, physical phenomena such as breakdown and impact ionization occur within the transistor, resulting in nonlinear effects [6,7,8]. In severe cases, thermal breakdown may occur inside the transistor. This can cause a sharp temperature rise, resulting in permanent damage to the LNA. This can cause the entire system to fail and become inoperable [9,10,11,12]. However, previous investigations have mainly focused on damage effects induced by HPM pulses with widths ranging from tens to hundreds of nanoseconds in GaAs pHEMT transistors or GaAs pHEMT LNAs. In fact, a limiter is connected to the front end of the LNA to protect it from interference caused by HPM [13,14,15]. This requires HPM with higher power to first damage the limiter before damaging the subsequent LNA.

In practice, the limiter requires a response time on the order of nanoseconds to protect the subsequent LNA [16,17]. Thus, high-power ultra-short microwave pulses with pulse widths of 1 ns or less can go through the limiter with a low attenuation before the limiter responds. This allows them to be directly injected into the LNA [18]. Most existing studies have investigated the generation of high-power ultra-short microwave pulses using passive pulse compression technologies. These include time-reversal (TR) technologies [19,20,21] and frequency-modulated (FM) pulse compression [22,23,24,25,26,27]. They can be used to generate microwave pulses with pulse widths in the sub-nanosecond range, peak power up to the GW range, and repetition rates up to tens of MHz [28]. Figure 1 shows a schematic illustration of LNA damage induced by high-power ultra-short microwave pulses. These pulses are coupled into the front end of the RF receiver, bypass the limiter, and directly damage the LNA.

This paper further analyzes the degradation and damage mechanisms of a GaAs-based pHEMT LNA under high-power mono-frequency ultra-short microwave injection. The results show that the negative half-cycle of the high-power microwave depletes the electron concentration in the transistor. As a result, ultra-short microwave with excessively high repetition rates are less effective in heating the device. However, an appropriate repetition rate allows the transistor sufficient time for the electron concentration to recover. Moreover, it can cause high-power ultra-short microwave pulses to rapidly degrade and burnout the LNA. These results provide a basis for damaging LNAs using FM pulse-compressed signals. This paper is organized as follows: Section 2 describes the modeling of the GaAs pHEMT transistor and LNA circuit using Technology Computer-Aided Design (TCAD). Section 3 analyzes the damage and degradation mechanisms of LNA under high-power mono-frequency ultra-short microwave pulse with varied power, repetition rate and frequency. In Section 4, experimental results of FM pulse compression signals will be presented. Section 5 presents the conclusion.

## 2. Modeling of GaAs-Based pHEMT LNA

The key component of the LNA studied in this paper is the GaAs pHEMT transistor inside it. Figure 2a shows the conventional structure of a GaAs-based pHEMT. The device consists, from bottom to top, of a GaAs substrate, an InGaAs channel, an AlGaAs spacer, a GaAs cap layer, and a Si_3_N_4_ passivation layer. The AlGaAs/InGaAs heterojunction forms a high-density two-dimensional electron gas (2DEG), enabling high-frequency and high-speed device operation. A 2-nm δ-doped structure is present in the AlGaAs spacer. This structure deepens the quantum well, thereby increasing the electron concentration in the 2-DEG. The specific dimensions of each component are shown in the figure.

Since this investigation focuses on damage effects, thermodynamic models are incorporated in addition to the Poisson equation and carrier transport equations [8,29,30]. In this case, the Electron current density ${\vec{J}}_{n}$ and hole current density ${\vec{J}}_{n}$ require the addition of a thermoelectric contribution term. The resulting equations can be written as follows:

(1) $$\begin{array}{c} {\vec{J}}_{n}=-qn\mu_{n}\left(\nabla \varphi_{n}+P_{n}\nabla T\right),\\ {\vec{J}}_{p}=-qn\mu_{p}\left(\nabla \varphi_{p}+P_{p}\nabla T\right), \end{array}$$

where $\mu_{n}$ and $\mu_{p}$ correspond to the mobilities of electrons and holes, respectively; $\nabla \varphi_{n}$ and $\nabla \varphi_{p}$ denote the quasi-Fermi potentials of electrons and holes; $P_{n}$ and $P_{p}$ represent the absolute thermoelectric powers of electrons and holes, respectively; and *T* denotes the temperature. The self-heating effect of the device is also considered and is described by the following equation:

(2) $$\begin{array}{cc} \frac{\partial }{\partial t}\left(c_{L}T\right)-\nabla \cdot \left(\kappa \nabla T\right) & =-\nabla \cdot \left[\left(P_{n}T+\varphi_{n}\right){\vec{J}}_{n}+\left(P_{p}T+\varphi_{p}\right){\vec{J}}_{p}\right]\\ \; & \;-\frac{1}{q}\left(E_{C}+\frac{3}{2}kT\right)\left(\nabla \cdot {\vec{J}}_{n}-qR_{\mathrm{net},n}\right)\\ \; & \;-\frac{1}{q}\left(-E_{V}+\frac{3}{2}kT\right)\left(-\nabla \cdot {\vec{J}}_{p}-qR_{\mathrm{net},p}\right)+\hbar \omega G^{\mathrm{opt}}. \end{array}$$

In this equation, κ denotes the thermal conductivity; $c_{L}$ represents the lattice heat capacity; $E_{C}$ and $E_{V}$ are the energies at the bottom of the conduction band and the top of the valence band, respectively; $G^{\mathrm{opt}}$ is the generation rate of photogenerated carriers produced by photons with angular frequency ω; $R_{\mathrm{net},n}$ and $R_{\mathrm{net},p}$ denote the net recombination rates of electrons and holes, respectively; and *ħ* is the reduced Planck constant.

In practice, the thermal conductivity and lattice heat capacity are not constants but vary with temperature. S. Adachi and J. S. Blakemore experimentally characterized the thermal conductivity and lattice heat capacity of GaAs [31,32]. Based on their experimental results, the corresponding GaAs material parameters used in this work were modified accordingly. The temperature-dependent expressions are given as follows:

(3) $$\begin{array}{cc} C(T) & =1.61+2.08\times 10^{-4}T+4.09\times 10^{-9}T^{2},\\ \kappa (T) & =0.67-9.75\times 10^{-5}T+3.89\times 10^{-9}T^{2}. \end{array}$$

A thermal interface resistance of $5\times 10^{-4}\;{\mathrm{cm}}^{2}\;K\;W^{-1}$ was also included, assuming negligible thermal resistance to the ambient environment. The initial lattice temperature was set to 300 K. Besides these, high-field electron mobility saturation and doping-dependent mobility in GaAs are also taken into account. Carrier generation and recombination are modeled using SRH, Auger, radiative, and electron-avalanche mechanisms. Donor-type traps are added in the GaAs substrate, and thermionic electron transport is factored in at the GaAs/AlGaAs heterointerface. These setups help capture how high-field carrier transport and the electrothermal behavior of the pHEMT interact under high-power ultra-short microwave signals.

Based on the device described above, an LNA circuit was constructed using TCAD mixed-mode simulation. The circuit was designed to operate primarily over the frequency range of 2–6 GHz, as shown in Figure 2b [29]. Among them, R_1_ and R_3_ are the series resistors between the DC supply and the drain, and between ground and the source, respectively. By adjusting the values of these two resistors, the pHEMT can be biased to different static operating points. R_2_ and R_4_ are the LNA input and output resistors, respectively. L_1_ and C_1_ form the input matching circuit, while L_3_ is the negative feedback inductor, together comprising the entire matching network. L_2_ is the DC feed inductor, which ensures that the DC signal is fed stably to the drain without being affected by the output signal. C_2_ is the output DC-blocking capacitor, which prevents the output signal from having a large DC offset. Ultimately, the transistor’s static operating point is V_*ds*_ = 2.5 V and V_*gs*_ = 0 V. The gain curve for this LNA is shown in Figure 2c. It achieves a gain above 8 dB over the 2–6 GHz range, with a maximum gain of 10.2 dB at 4.8 GHz.

## 3. Analysis of LNA Failure Mechanisms Under Ultra-Short Microwave Pulses

**Figure 3:**
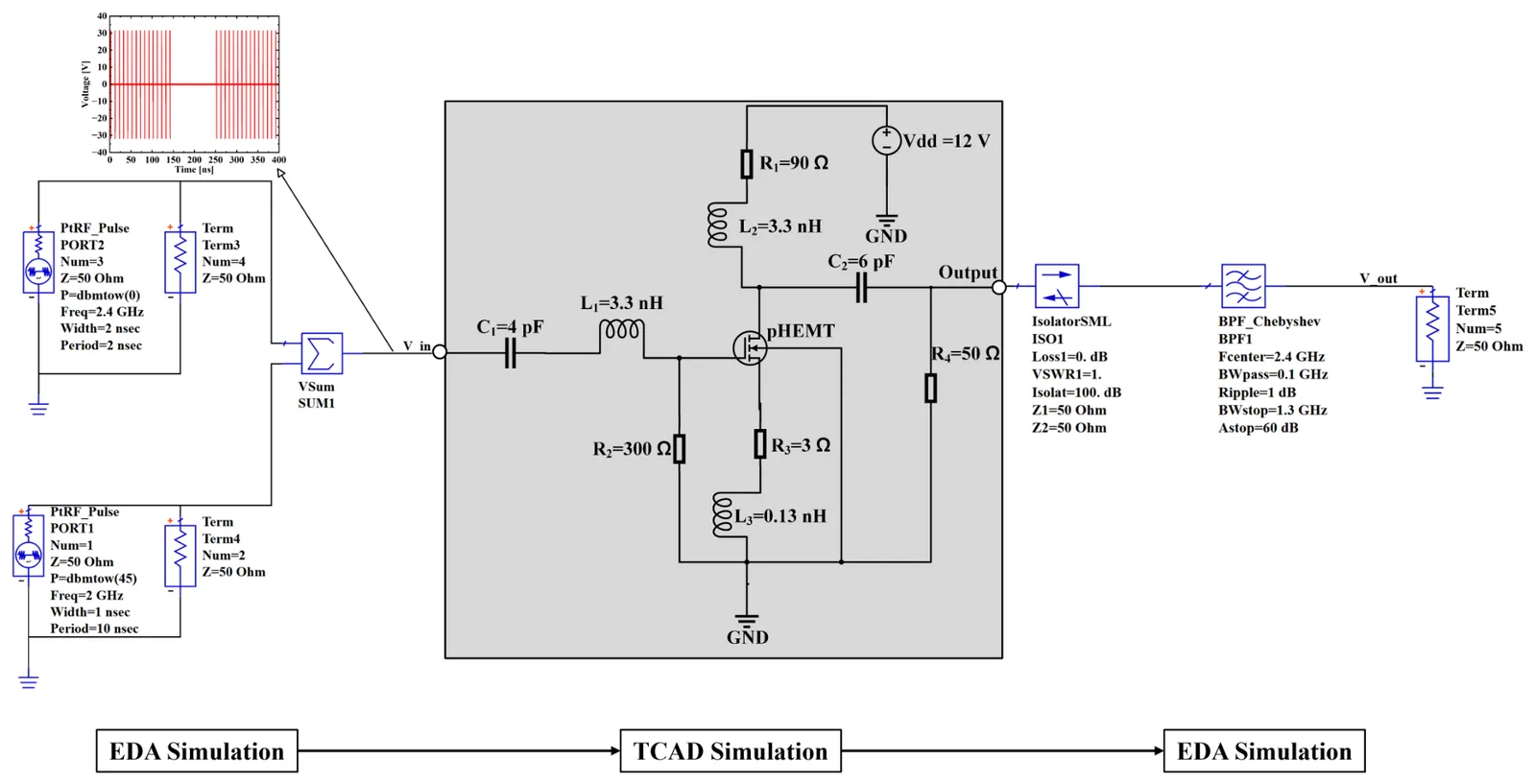
Schematic diagram of the EDA and TCAD co-simulation.

Figure 3 shows the circuit diagram for this simulation. Electronic Design Automation (EDA) software was used to generate both a 2.4 GHz low-power signal and a 2 GHz high-power ultra-short microwave pulse, which were then combined using a power combiner. The frequency of 2.4 GHz is selected because it lies within the operating band of the investigated LNA and is also a typical ISM-band frequency widely used in microwave and wireless systems. Therefore, it has strong practical relevance for evaluating the susceptibility of RF front-end circuits to high-power microwave pulses.

The high-power ultra-short microwave pulses have a repetition rate of 100 MHz and are injected with a 2 ns delay. This allows the LNA to operate normally for 2 ns. After 150 ns pulses injecting, the injection was paused for 100 ns and then resumed to observe the recovery of the LNA. The output signal from the LNA will then be fed back into EDA, where a filter will remove the high-power signal, leaving only the 2.4 GHz low-power signal. High-power ultra-short microwave pulses at 2.4 GHz are not used here or in the following sections. This is because the filter cannot distinguish between high-power interference signals at 2.4 GHz and low-power normal signals. An isolator is used to prevent reflections from the filter from affecting the signal input. The following subsections examine the effects of pulse power, repetition rate, and frequency of ultra-short microwave pulses on the temperature and gain of the LNA.

### 3.1. The Effect of Ultra-Short Microwave Pulse Power on the LNA

**Figure 4:**
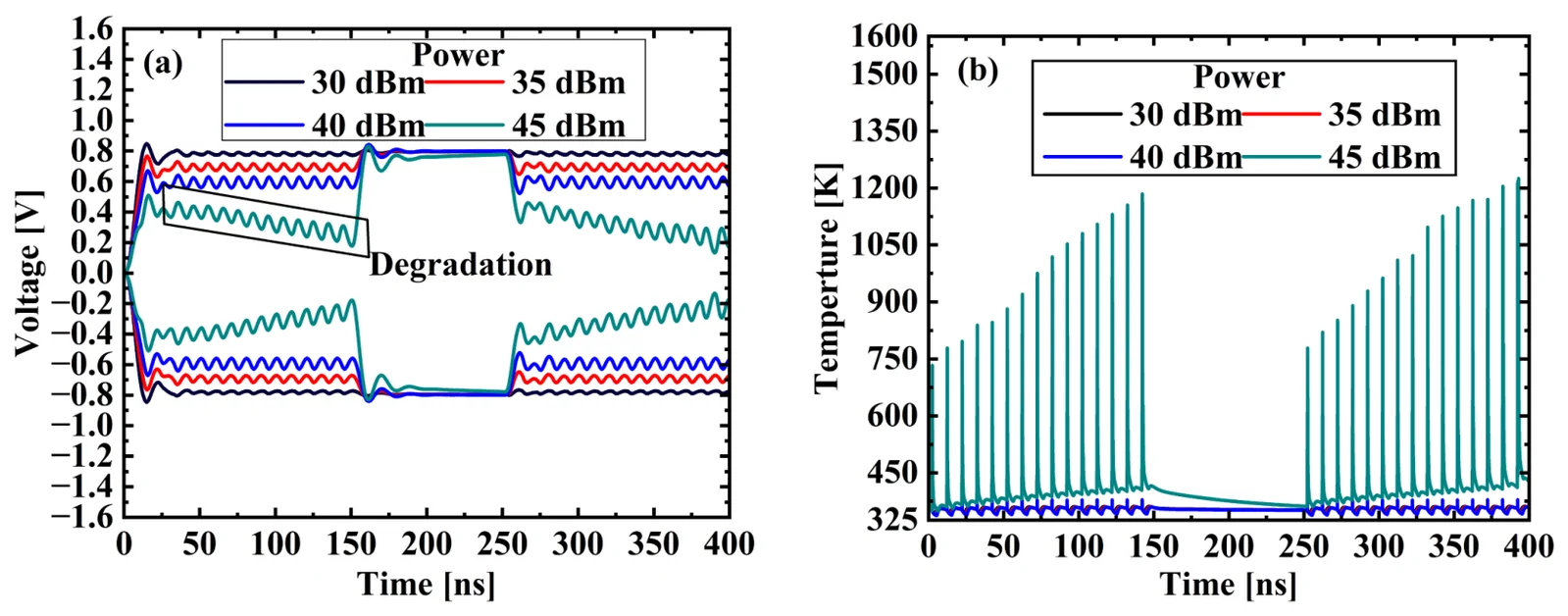
The effect of power of ultra-short microwave pulses on (**a**) signal interference and (**b**) transistor temperature.

The LNA investigated in this study primarily operates at 2–6 GHz. When studying the effect of power on damage to the LNA, 2 GHz is used first. This will be increased to 5 GHz in the following subsections. This simulation uses high-power ultra-short mono-frequency microwave pulses to degrade and damage the LNA. This is because EDA can easily generate such signals. In fact, the pulse compression signals will be used in subsequent experiments caused damage and degradation to the LNA. Thus Figure 4 shows the output signals and temperature of the transistor under ultra-short microwave pulses with power of 30, 35, 40 and 45 dBm, frequency of 2 GHz and pulse width of 1 ns (two full cycles) injecting. It can be seen that the amplification of the 2.4 GHz signal by the LNA is intermittently suppressed and restored. Furthermore, as the power of ultra-short microwave pulses increases, the degree of interference experienced by the LNA gradually increases. This is caused by the continuous depletion and recombination of electrons within the transistor due to the ultra-short microwave pulses. As the input power increased from 30 to 40 dBm, the maximum temperature inside the transistor rose only from 360 to 378 K, which was insufficient to cause significant heat buildup. Furthermore, when the power reached 45 dBm, the LNA began to degrade gradually. The gain of the LNA drops to −2 dB at 142.5 ns. At the same time, the maximum temperature inside the transistor rose to 1160 K. However, when the injection of high-power ultra-short microwave pulses was stopped between 150 ns and 250 ns, the gain of the LNA returned to approximately 7.9 dB.

**Figure 5:**
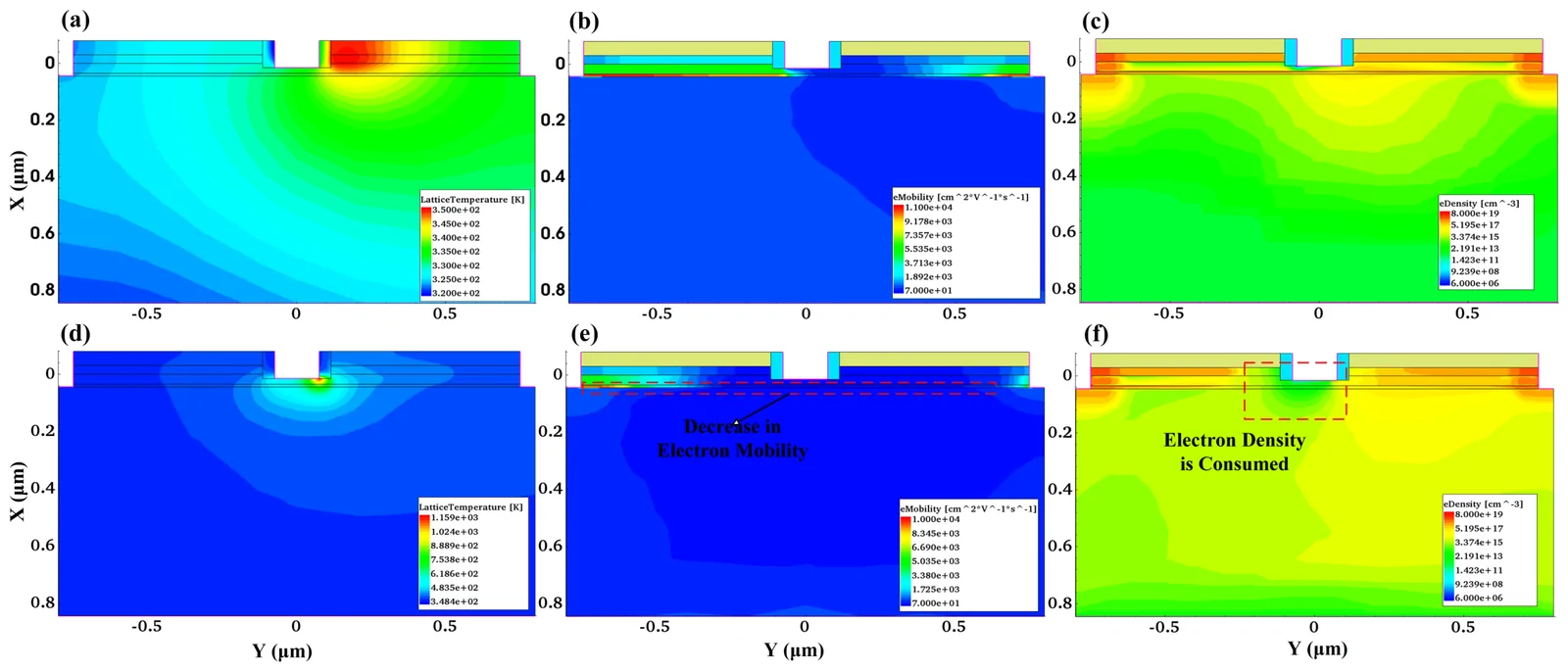
Comparison of physical parameters in transistors under normal operation and high-power ultrashort microwave injection (**a**) lattice temperature, (**b**) electric mobility and (**c**) electric density at 1 ns; (**d**) lattice temperature, (**e**) electric mobility and (**f**) electric density at 142.5 ns.

To identify the cause of the above issue, the physical parameters of the transistor at 142.5 ns were compared with those during normal operation at 1 ns, as shown in Figure 5. The effect of high-power ultra-short microwave pulses on the electron density in transistors can be seen by comparing Figure 5c and Figure 5f. The negative half-cycle of the high-power ultra-short microwave pulse causes the electron concentration beneath the transistor’s gate to be depleted. This explains why the amplification of the 2.4 GHz signal is intermittently suppressed and restored. Moreover, when the power of ultra-short microwave pulses reaches 45 dBm, heat can accumulate inside the transistor. The electron mobility in the transistor’s conduction channel decreases (Figure 5e) as the temperature rises (Figure 5d). This is the cause of transistor degradation. However, when the injection of ultra-short microwave pulses stops, the temperature inside the transistor gradually decreases [33,34]. This will restore the electron mobility of the transistor. As can be seen in Figure 4, the gain of the LNA gradually recovers from 150 ns to 250 ns. The gain recovers to 7.1 dB at 200 ns and to 7.9 dB at 250 ns. As can be seen, the degrading effects of high-power ultra-short microwave pulses on LNA primarily affect the electron density and mobility of the transistors. Furthermore, all of these effects are reversible before the transistor burnout.

**Figure 6:**
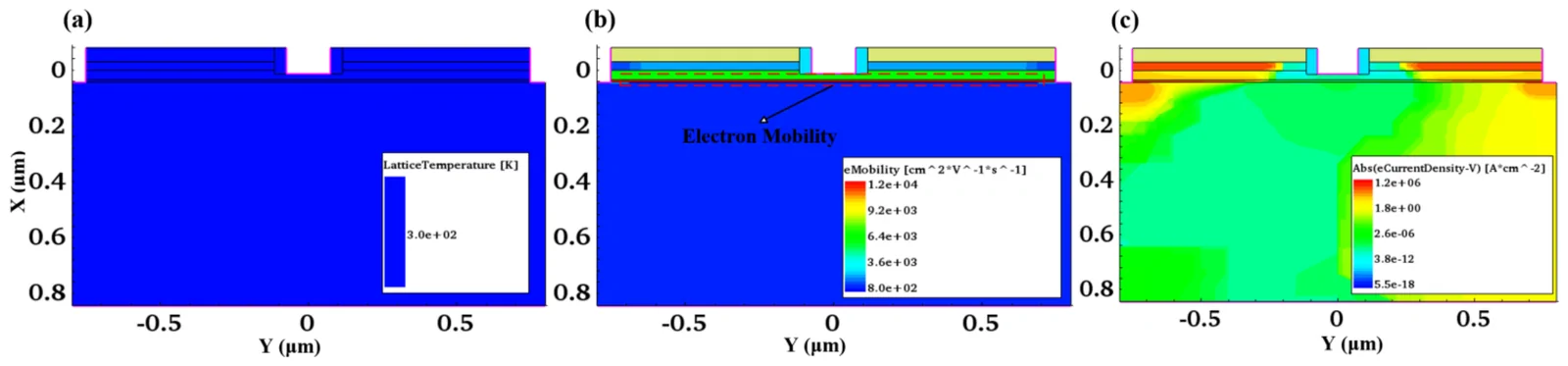
Physical state of the transistor at 142.5 ns with the self-heating model disabled (**a**) lattice temperature, (**b**) electric mobility and (**c**) electric density.

To further demonstrate that the degradation in LNA performance is primarily caused by the temperature rise, the self-heating model in Equation (2) is disabled. The lattice temperature, electron mobility and electron density at 142.5 ns with the self-heating model disabled are shown in Figure 6. Compared with the case in which self-heating is enabled, the electron mobility exhibits no significant variation because the lattice temperature is maintained at 300 K. Meanwhile, the electron density remains relatively low, since the device temperature does not approach approximately 750 K [29,35], where intrinsic carrier excitation becomes much more pronounced in our simulation.

**Figure 7:**
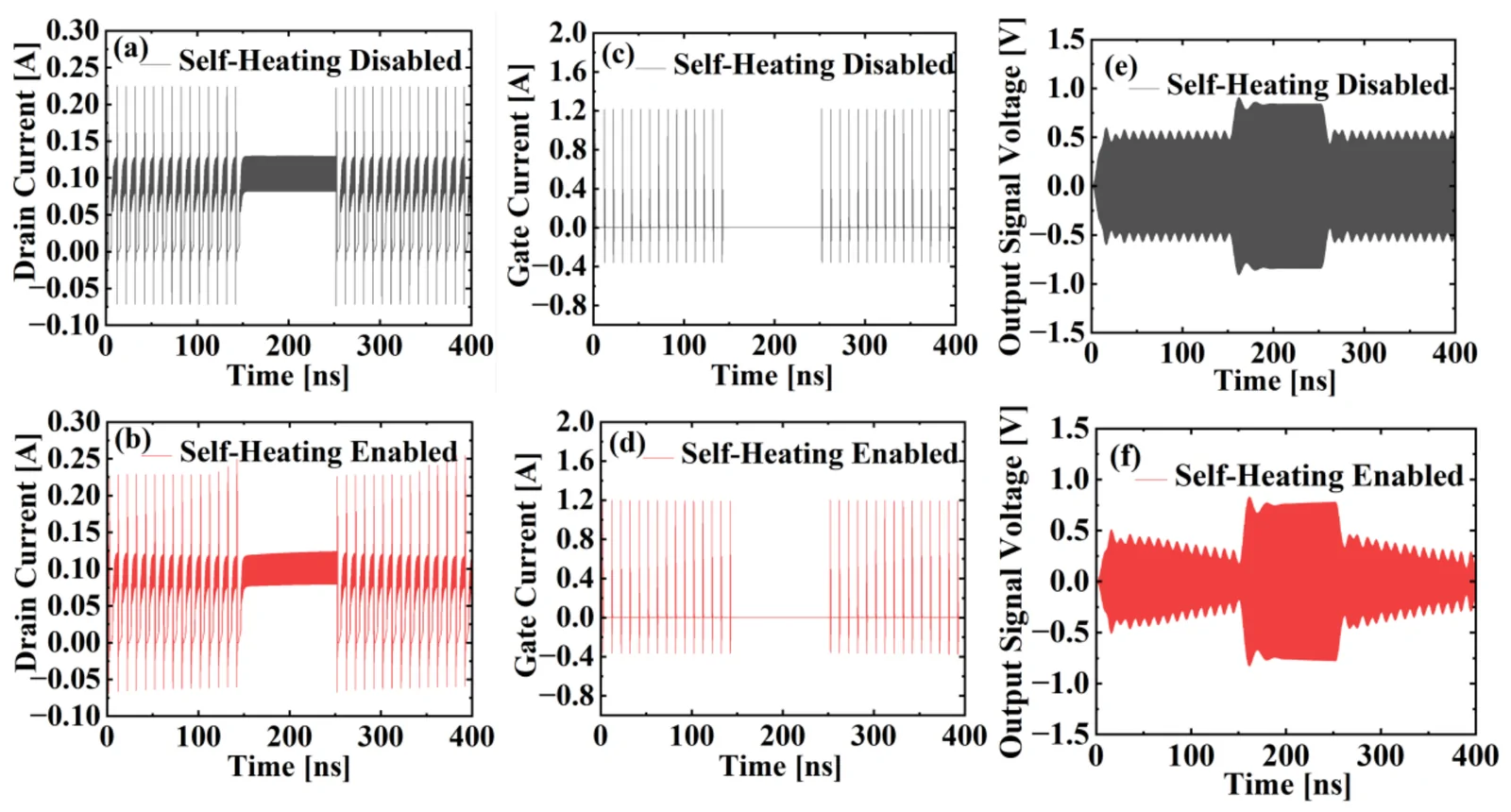
With the self-heating model disabled: (**a**) drain current, (**c**) gate current, and (**e**) output signal; with the self-heating model enabled (**b**) drain current, (**d**) gate current, and (**f**) output signal.

The corresponding drain current, gate current, and LNA gain are presented in Figure 7. When the self-heating model is disabled, the drain current of the transistor does not exhibit the degradation-and-recovery behavior observed when self-heating is enabled, while the gate current shows no significant variation. Moreover, the output signal after filtering does not exhibit any noticeable degradation. The comparison between simulations with and without self-heating further supports the interpretation that the increase in device temperature leads to a reduction in electron mobility, which subsequently contributes to the degradation of the LNA gain. We have revised related discussion in the text.

### 3.2. The Effect of Ultra-Short Microwave Pulse Frequency on the LNA

**Figure 8:**
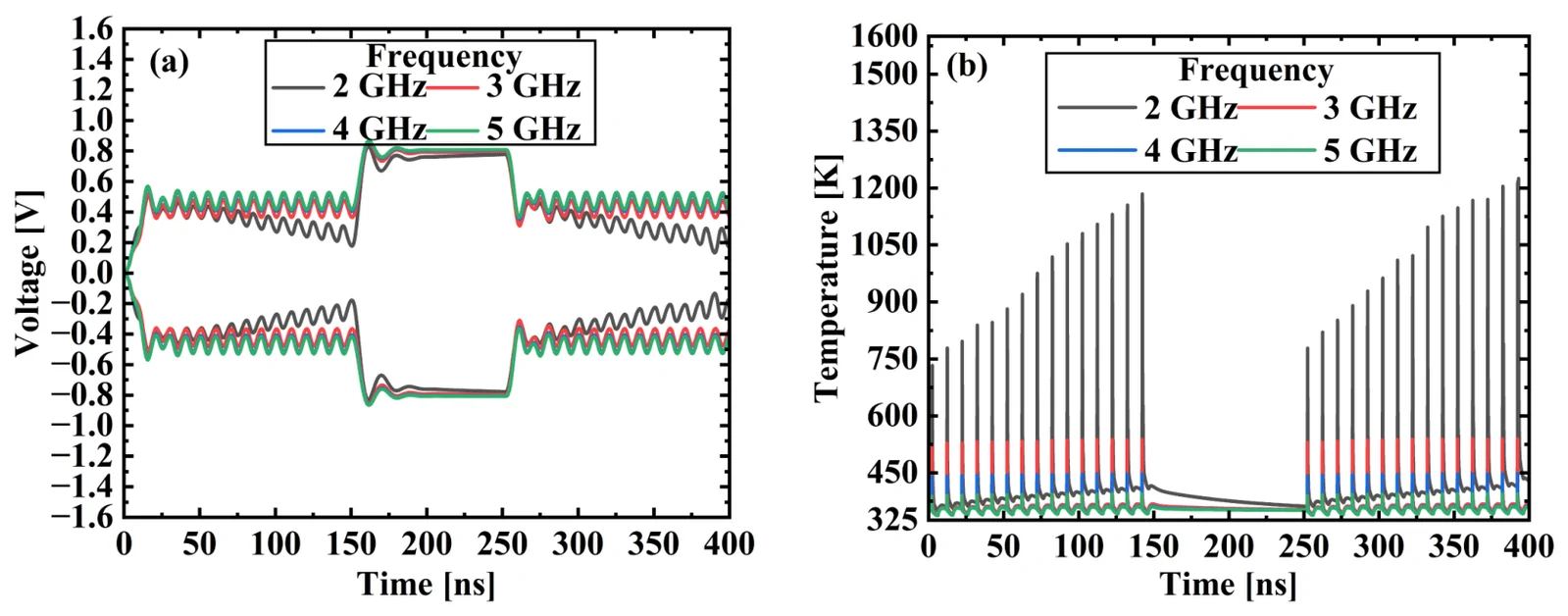
The effect of frequency of ultra-short microwave on (**a**) signal interference and (**b**) transistor temperature.

Figure 8 shows the interference effects of high-power ultrashort microwave pulses with a power of 45 dBm, a pulse width of 1 ns, a repetition rate of 100 MHz, and a frequency range of 2–5 GHz on the LNA. During the duration of high-power ultra-short microwave pulses, the gain decreased to −2, 2, 2.8 and 3.1 dB at 142.5 ns, respectively. No gradual decrease in gain is observed when the frequency in the 3–5 GHz range. This is for the reason that as the frequency increases, the amount of time it takes for heat generated by the transistor to accumulate decreases with each cycle. Ultra-short microwave pulses of 45 dBm at 3, 4 and 5 can only raise the transistor temperature to a maximum of 538, 447 and 394 K, respectively.

It is not possible to continuously reduce the electron mobility of the transistor. However, temperature still affects electron mobility. Therefore, the higher the frequency, the less effective the suppression of the gain of LNA. But the main reason is a decrease in electron density causes a drop in gain. Therefore, subsequent investigations into the effect of repetition frequency on the LNA continue to use high-power ultra-short microwave pulses at 2 GHz.

### 3.3. The Effect of Ultra-Short Microwave Pulse Repetition Rate on the LNA

**Figure 9:**
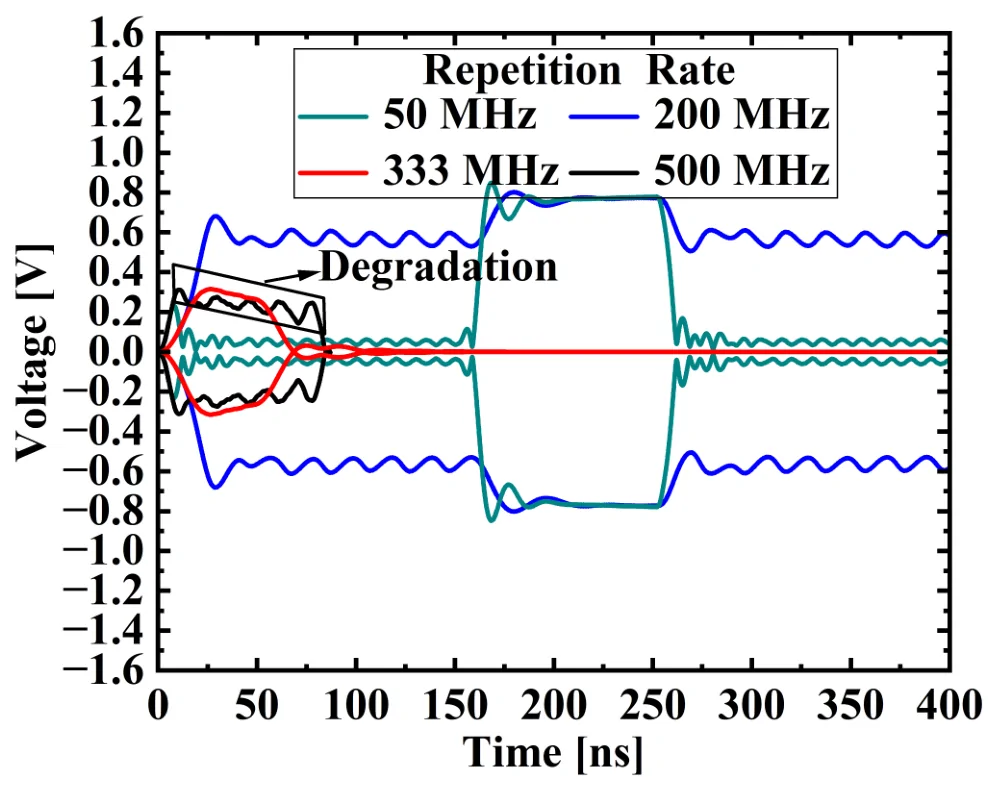
The effect of ultra-short microwave pulses at 2 GHz with different repetition rate on LNA amplifying 0 dBm, 2.4 GHz signal.

Figure 9 shows the effect of 45 dBm, 2 GHz ultra-short microwave pulses with different repetition rates on the output signal. As can be seen, as the repetition rate increases from 50 MHz to 500 MHz, the gain for the 2.4 GHz signal decreases from 4.5 dB to –18 dB. The interference with the signal therefore becomes stronger. This is because a higher repetition rate of high-power ultrashort microwave pulses has a more pronounced effect on the electron concentration within the transistor. However, the gradual degradation in gain was observed only at repetition rates of 200 MHz and 333 MHz and the subsequent signal disappeared. This is due to the effect of the internal temperature of the transistor. The transistors reached the melting temperature of GaAs (1511 K) at 43 ns and 87 ns, respectively. Thus the simulation is terminated.

**Figure 10:**
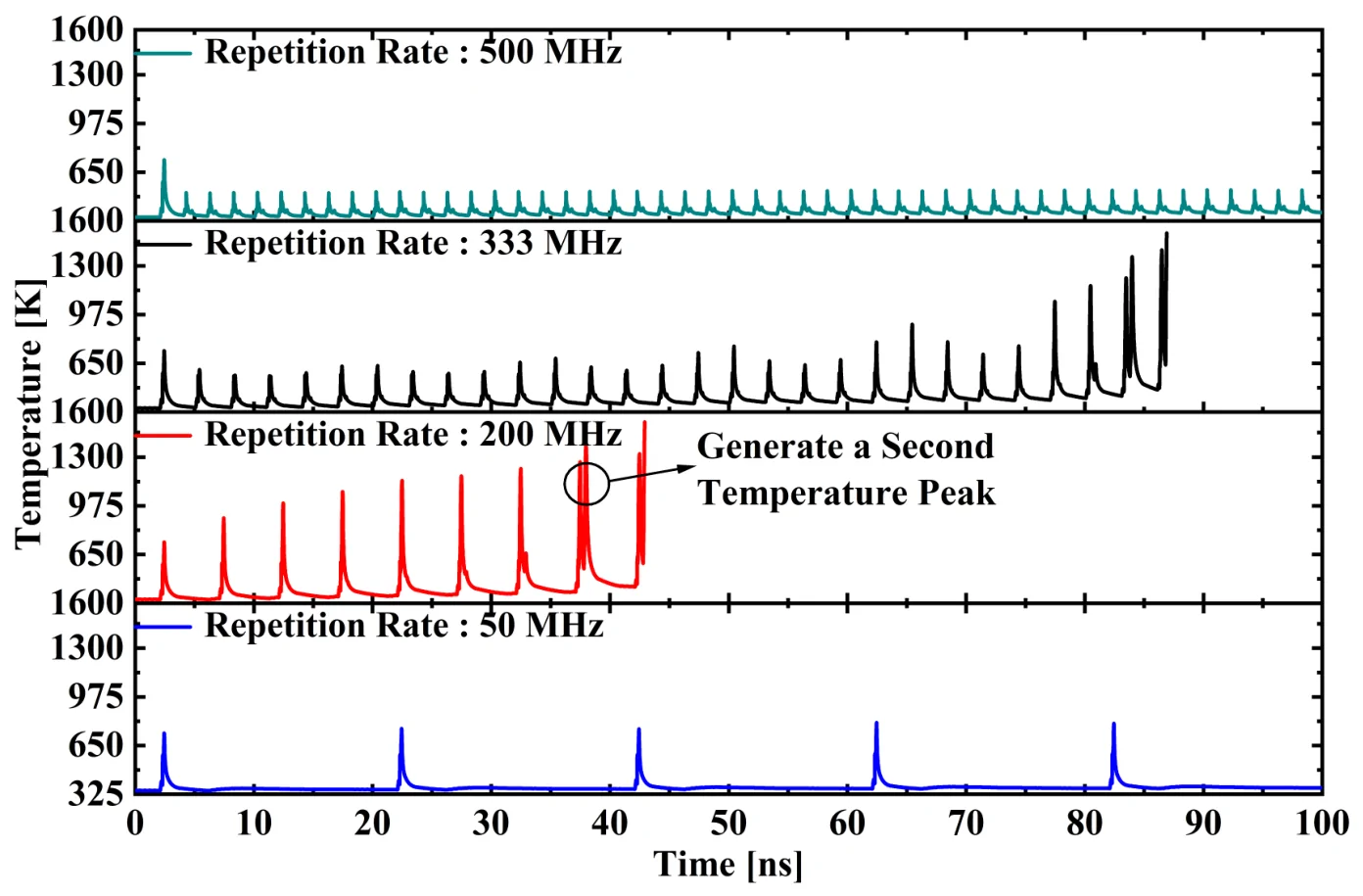
The effect of ultra-short microwave pulse repetition rate on transistor temperature.

Figure 10 shows the lattice temperature rise of the transistor at these four repetition rates. As can be seen, when the repetition rate is 50 MHz, the frequency is too low. This allows heat to dissipate within the transistor, preventing heat accumulation. However, when the repetition rate exceeds 500 MHz, only the temperature of the first cycle reaches 733 K. The transistor fails to heat up effectively during the subsequent cycles. This is because the first negative half-cycle of the high-power ultra-short microwave pulse significantly depletes the electron concentration within the transistor. This results in insufficient electron density in subsequent cycles to generate a current and thus produce heat. However, when the repetition rate is 200 MHz and 333 MHz, the heat inside the transistor has not yet dissipated, and the electrons inside have some time to recombine. This ultimately results in effective heat accumulation. When the internal temperature of the transistor reaches the intrinsic excitation temperature of GaAs (750 K), thermal breakdown occurs within the transistor. This results in the generation of a large number of electrons. This ensures that there is sufficient electron density in subsequent cycles to generate a current, which in turn produces a second peak temperature. Consequently, this region reached the melting temperature of GaAs (1511 K) [29,30] first at 43 and 87 ns, respectively, at these two repetition rates.

**Figure 11:**
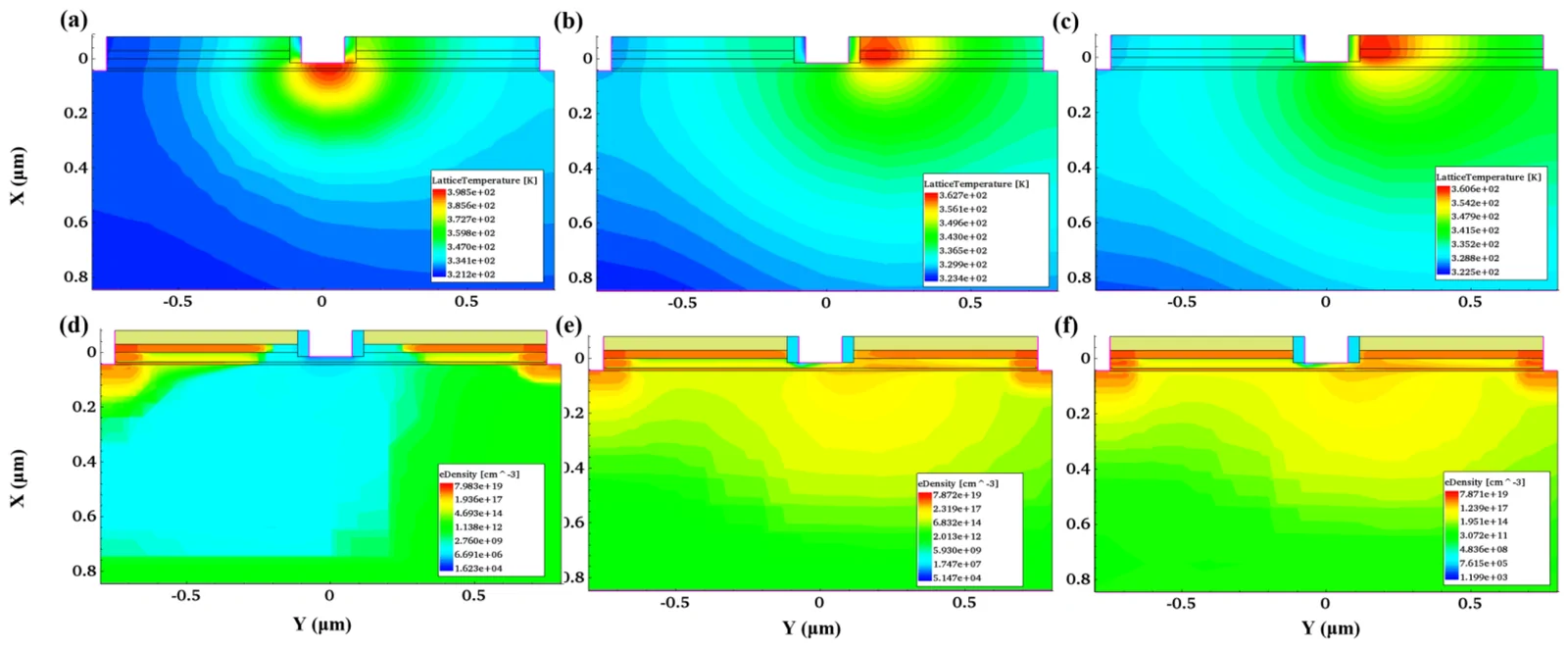
The lattice temperature of transistor at (**a**) 3 ns (**b**) 8 ns and (**c**) 12 ns; the electron density of transistor at (**d**) 3 ns (**e**) 8 ns and (**f**) 12 ns.

For a more intuitive observation of the heat dissipation and electron-density recovery in the transistor, the lattice temperature and electron density at 3 ns, 8 ns, and 12 ns are compared as shown in Figure 11. These time points correspond to the end of the first pulse (3 ns), the interval between the two pulses (8 ns), and the moment immediately before the arrival of the second pulse (12 ns), respectively, under the aforementioned repetition frequency of 100 MHz. It can see that from 3 ns to 8 ns, the temperature inside the transistor drops from 398 K to 362 K and shifts toward the drain. Meanwhile, the electron density under the gate also returns to a higher level. From 8 ns to 12 ns, the temperature inside the transistor slowly drops to 360 K, while the electron density doesn’t change much. That is, the electron density within the transistor can largely recover to its initial level within approximately 5 ns, thereby allowing the device to accommodate the subsequent injection of a high-power ultra-short microwave pulse.

**Figure 12:**
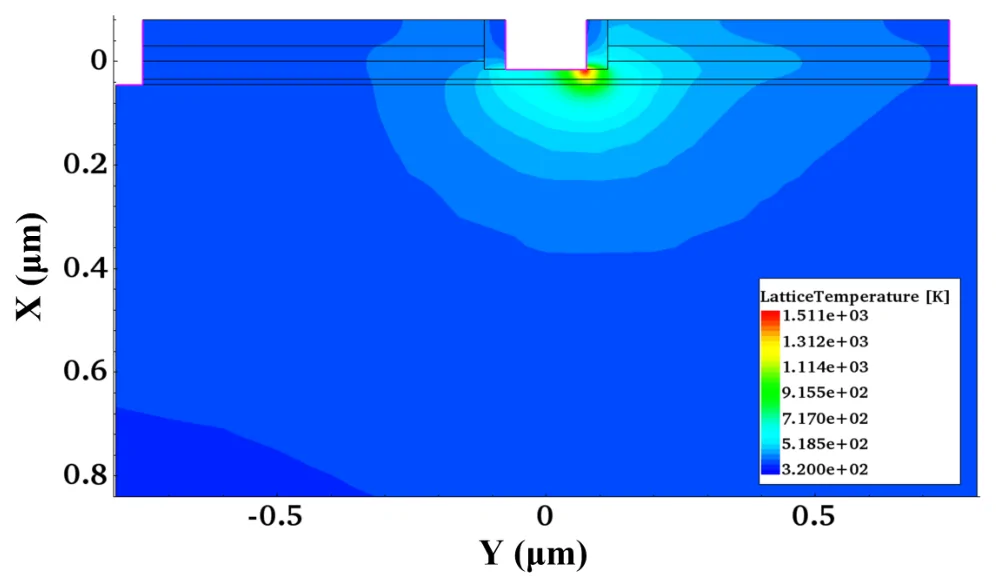
Lattice temperature of the burnout transistor.

Ultimately, the transistor burned out at a location below the gate, toward the drain side as shown in Figure 12. This is because the temperature rise in the transistor caused by high-power ultra-short microwaves occurs primarily during the negative half-cycle. The bias voltage present at the drain causes a larger potential difference between the gate and the drain. This ultimately caused that area to reach the burn-out temperature of GaAs first.

## 4. Experiment on the Generation of Ultra-Short Microwave Pulses

**Figure 13:**
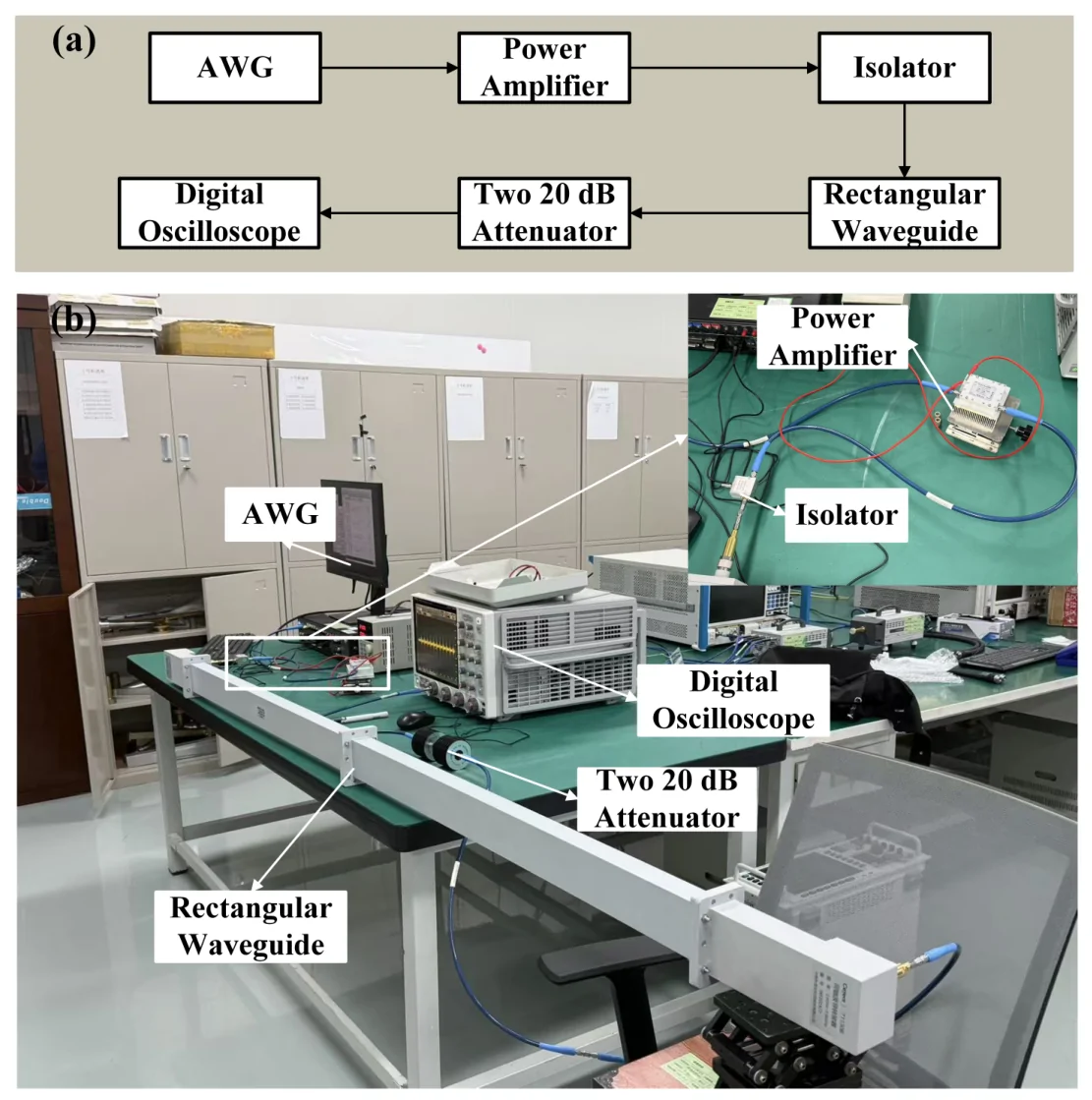
Experimental setup for generating high-power ultra-short microwave pulses (**a**) schematic diagram and (**b**) experimental setup.

The simulations described above were performed using high-power, mono-frequency ultra-short microwave pulses. In actual experiments, it is not easy to generate microwaves of such high power. Microwave pulses capable of generating such high power have pulse widths of several ns, which do not reach the sub-nanosecond range [36]. However, this can be easily achieved using FM pulse compression technology. The specific generation method is as follows: FM pulse compression is achieved by using a dispersive transmission line. In this type of transmission line, the group velocity of the electromagnetic wave depends on frequency. The input pulse is swept from a frequency component with a lower group velocity to one with a higher group velocity. As a result, the rear part of the pulse travels faster than the front part. It gradually catches up with the front part. This process shortens the pulse width and increases the pulse amplitude. Smooth straight waveguides can be used as dispersive transmission lines for pulse compression. They are often selected because of their simple structure. In this paper, a rectangular waveguide is used as a compressor and the specific experimental setup is shown in Figure 13. Since the frequency range of the aforementioned simulation study is 2–6 GHz, an arbitrary waveform generator (AWG) with a sample rate of 65 GSa/s is used to generate an FM pulse with a frequency range from 2.182 to 5.182 GHz, a pulse width of 18 ns, and a power of 0 dBm. It is then amplified by the power amplifier (PA) with an operating frequency of 0.05–6 GHz to 33 dBm. Next, the pulses are compressed through a 2 m long aluminum rectangular waveguide WR284 (72.14 × 34.04 mm^2^). The isolator and two attenuators are used to protect the PA and the digital oscilloscope, respectively.

**Figure 14:**
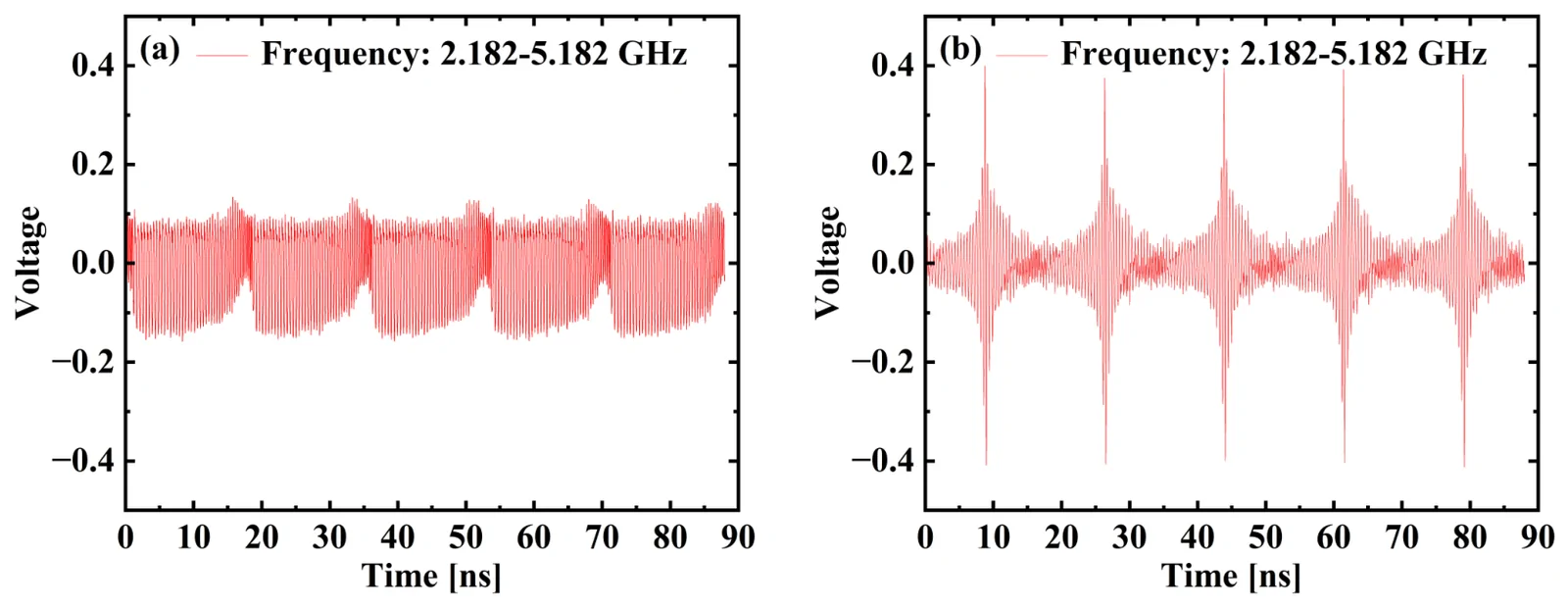
Comparison of pulse compression signal waveforms before and after compression (**a**) waveform before compression, (**b**) waveform after compression.

The compressed pulses generated by this method are shown in Figure 14. The compressed pulses achieve a power compression factor of 6.4 and a peak power of 42 dBm. Its 3-dB pulse width is 0.57 ns, and the repetition rate reaches up to 56 MHz. Since the saturated output of the PA is only 33 dBm, this limits the peak power of the final compressed pulses. In fact, using a PA with higher output power can result in a higher peak power up to several kW for the compressed pulses. Future work will investigate LNA degradation and damage using high-power ultra-short FM microwave pulses.

## 5. Conclusions

In this paper, we use a co-simulation of TCAD and EDA to analyze the degradation and damage mechanisms caused by high-power ultra-short microwave pulses on the GaAs-based pHEMT LNA. The simulation results show that, because the negative half-cycle of the HPM depletes the electron concentration in the transistor, a 45 dBm ultra-short microwave pulses with excessively high repetition rates (the maximum is a continuous wave) at 2 GHz does not readily induce rapid transistor burnout. However, ultra-short microwave pulses with a repetition rate of 200 MHz can burn out the transistor within 43 ns because this repetition rate allows sufficient time for the electron concentration in the transistor to partially recover between adjacent pulses. Furthermore, the temperature rise reduces the electron mobility in the transistor, while the injection of high-power ultra-short microwave pulses causes an imbalance in electron concentration. These two effects jointly degrade transistor performance and ultimately reduce the gain of the LNA. Moreover, experiments can generate ultra-short microwave pulses with durations of 0.53 ns, peak powers of 42 dBm, and repetition rates of 56 MHz by using FM pulse compression technology. Although damage experiments on the LNA under high-power ultra-short microwave pulse excitation have not yet been performed, the present experimental results confirm the feasibility of the pulse-generation approach and support its potential application in subsequent LNA damage studies.

## Data Availability

The original contributions presented in this study are included in the article. Further inquiries can be directed to the corresponding author.
